# Inflammatory and Pro-resolving Lipids in Trypanosomatid Infections: A Key to Understanding Parasite Control

**DOI:** 10.3389/fmicb.2018.01961

**Published:** 2018-08-21

**Authors:** Rodrigo A. López-Muñoz, Alfredo Molina-Berríos, Carolina Campos-Estrada, Patricio Abarca-Sanhueza, Luis Urrutia-Llancaqueo, Miguel Peña-Espinoza, Juan D. Maya

**Affiliations:** ^1^Instituto de Farmacología y Morfofisiología, Facultad de Ciencias Veterinarias, Universidad Austral de Chile, Valdivia, Chile; ^2^Instituto de Investigación en Ciencias Odontológicas, Facultad de Odontología, Universidad de Chile, Santiago, Chile; ^3^Escuela de Química y Farmacia, Facultad de Farmacia, Universidad de Valparaíso, Valparaíso, Chile; ^4^Centro de Investigación Farmacopea Chilena, Universidad de Valparaíso, Valparaíso, Chile; ^5^Programa de Farmacología Molecular y Clínica, Instituto de Ciencias Biomédicas, Facultad de Medicina, Universidad de Chile, Santiago, Chile

**Keywords:** resolution of inflammation, prostaglandins, leukotrienes, resolvins, lipoxins, *Trypanosoma cruzi*, *Trypanosoma brucei* spp., *Leishmania* spp.

## Abstract

Pathogenic trypanosomatids (*Trypanosoma cruzi, Trypanosoma brucei*, and *Leishmania* spp.) are protozoan parasites that cause neglected diseases affecting millions of people in Africa, Asia, and the Americas. In the process of infection, trypanosomatids evade and survive the immune system attack, which can lead to a chronic inflammatory state that induces cumulative damage, often killing the host in the long term. The immune mediators involved in this process are not entirely understood. Most of the research on the immunologic control of protozoan infections has been focused on acute inflammation. Nevertheless, when this process is not terminated adequately, permanent damage to the inflamed tissue may ensue. Recently, a second process, called resolution of inflammation, has been proposed to be a pivotal process in the control of parasite burden and establishment of chronic infection. Resolution of inflammation is an active process that promotes the normal function of injured or infected tissues. Several mediators are involved in this process, including eicosanoid-derived lipids, cytokines such as transforming growth factor (TGF)-β and interleukin (IL)-10, and other proteins such as Annexin-V. For example, during *T. cruzi* infection, pro-resolving lipids such as 15-epi-lipoxin-A4 and Resolvin D1 have been associated with a decrease in the inflammatory changes observed in experimental chronic heart disease, reducing inflammation and fibrosis, and increasing host survival. Furthermore, Resolvin D1 modulates the immune response in cells of patients with Chagas disease. In *Leishmania* spp. infections, pro-resolving mediators such as Annexin-V, lipoxins, and Resolvin D1 are related to the modulation of cutaneous manifestation of the disease. However, these mediators seem to have different roles in visceral or cutaneous leishmaniasis. Finally, although *T. brucei* infections are less well studied in terms of their relationship with inflammation, it has been found that arachidonic acid-derived lipids act as key regulators of the host immune response and parasite burden. Also, cytokines such as IL-10 and TGF-β may be related to increased infection. Knowledge about the inflammation resolution process is necessary to understand the host–parasite interplay, but it also offers an interesting opportunity to improve the current therapies, aiming to reduce the detrimental state induced by chronic protozoan infections.

## Overview of Arachidonic Acid Metabolism and the Pro-Resolving Lipid Mediators

Inflammation is a pathophysiologic process that occurs in the context of broad spectra of stimuli and diseases including arthritis, asthma, trauma, and infection. During acute infection, inflammation is protective, but if it is excessive or prolonged, it harms the host, damaging tissues and impairing proper repair, and in extreme cases, it can be lethal. Repair and restoration of normal organ function are essential after an infectious disease, and these processes are accomplished after the inflammatory events are appropriately resolved. However, resolution of inflammation is a more intricate process than the mere dissipation of chemoattractant signals. It includes a set of complex events mediated by several signals, including negative feedback regulation of Toll-like receptor (TLR) signaling, production of anti-inflammatory cytokines such as interleukin (IL)-10, and biosynthesis of a superclass of novel mediators. These newly discovered mediators include biochemical species derived from lipids such as lipoxins (LXs), resolvins (Rvs), protectins (PDs), and maresins (Serhan, 2005), proteins such as Annexin A1 (Sugimoto et al., 2016) and Galectin-1 (Sundblad et al., 2017), anti-inflammatory neuropeptides such as melanocortin (MC) peptide (Delgado and Ganea, 2008; Alessandri et al., 2013), and gasotransmitters such as hydrogen sulfide and carbon monoxide (Wallace et al., 2015; Shinohara and Serhan, 2016). The concerted actions of these molecules stop leukocyte recruitment, modify cytokine production, facilitate efferocytosis, switch macrophages to a non-phlogistic phenotype, and finally, promote healing to restore organ function (Serhan, 2014).

**FIGURE 1:**
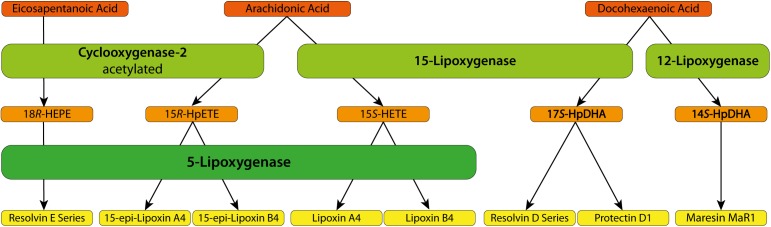
Biosynthetic pathways of specialized pro-resolving mediators.

Specialized pro-resolving mediators (SPMs), including the pro-resolving lipids, are produced via cell–cell interactions within the inflammatory exudates that control the magnitude and duration of local inflammation (Serhan and Chiang, 2013). SPMs are all products of the lipoxygenase (LO) pathway, though the lipid substrates vary (**Figure 1**).

### SPMs Synthesis

#### Lipoxins

Lipoxins are eicosanoids derived from omega-6 arachidonic acid (AA), which is oxygenated at position 15 by 15-LO activity to produce 15S-hydroperoxyeicosatetraenoic acid (15S-H(p)ETE). The product of 5-LO’s action on 15-HpETE is a 5S-hydroperoxy,15S-hydro(peroxy)-DiH(p)ETE, which is rapidly converted to 5(6)-epoxytetraene. In turn, 5(6)-epoxytetraene is rapidly hydrolyzed to lipoxin A4 (LXA_4_) and LXB_4_ (Serhan, 2005). Alternatively, acetylsalicylic acid (ASA)-acetylated cyclooxygenase 2 (COX-2) produces 5-*R*-Hydroxyeicosatetraenoic acid (5-*R*HETE) from AA. 5-*R*HETE is a substrate of 5-LO that can be converted to 15-epi-lipoxin A4 (15-epi-LXA_4_), which is also named ASA-triggered lipoxin (ATL) (Serhan, 2005).

#### Resolvins

On the other hand, Rvs, PDs, and maresins are derived from omega-3 polyunsaturated fatty acids. There are two series of Rvs depending on the lipid substrate and enzyme activities: Rv D and E. The RvD1–4 series, as well as PDs and maresins, are derived from docosahexaenoic acid (DHA) metabolism involving 12-LO and 15-LO, and the E-series Rvs are derived from the activity of ASA-acetylated COX-2 using eicosapentaenoic acid (EPA) as a substrate (Serhan, 2007). Nevertheless, ASA can also trigger the COX-2-mediated conversion of DHA to 17R-HDHA, which 5-LO can covert to ASA-triggered Resolvin D1–4 (AT-RvD1–4) (Serhan, 2007).

### Drug Induction of SPMs

As mentioned, ASA modifies COX-2 activity allowing 15-epi-LXA4 and AT-RvD1–4 production. Indeed, ASA-triggered resolving lipids are more stable than the endogenous molecules and could serve as anti-inflammatory drugs. Another fascinating group of drugs, statins, can also increase the production of pro-resolving lipids, in a way that is similar to the process mediated by ASA. Just as the acetylation of COX-2 by ASA causes a metabolic switch in COX-2, statins produce the same metabolic switch by nitrosylation. Recently, Serhan’s group reported the presence of another subtype of Rvs, 13-series Rvs, which provide protective activity against *Escherichia coli* infections. These lipids are produced by COX-2 in a process involving a neutrophil–endothelium interaction, and their production can also be triggered by nitrosylation induced by atorvastatin (Dalli et al., 2015). Thus, the change in COX-2 activity explains, at least in part, the anti-inflammatory properties of ASA and statins.

### The Inflammation Resolution Crossroad

It is remarkable how the same enzymatic array participates in the generation of both inflammatory and resolving mediators. COX and LO activities, which are responsible for the production of prostaglandins (PGs) and leukotrienes, respectively, can switch to the production of LX, Rvs, PDs, and maresins. Indeed, AA derivatives, PGs E_2_ (PGE_2_), prostacyclin (PGs I2, PGI_2_), and leukotriene B_4_ (LTB_4_), participate in leukocyte recruitment to the damaged site. However, as acute inflammation progresses, a metabolic switch occurs, and LX synthesis begins. The exact moment when this switch happens is unclear; however, the dampening of the inflammatory signals may be part of the input needed to promote the metabolic changes (Gilroy and De Maeyer, 2015). These switches are accomplished by transcriptional or posttranslational modifications, which involve PGE_2_ and PGs D_2_ (PGD_2_) (Frolov et al., 2013; Croasdell et al., 2015). COX-mediated production of PGD_2_ by human PGD_2_ synthase (hPGD_2_s) activates the PGD_2_ receptor, DP_1_ (a G-protein coupled receptor [GPCR]), which in turn stimulates the production of IL-10 (an anti-inflammatory cytokine), which then blocks the path to chronic inflammation. PGD_2_ can also be converted to PGs J2 (PGJ_2_) and 15-deoxy-Δ(12,14)-PGs J_2_ (15-D-PGJ_2_) products that activate peroxisome proliferator-activated receptor (PPAR)-γ to promote resolution (Croasdell et al., 2015).

### SPMs Mode of Action

The actions of LXA_4_ are mediated by a GPCR called formyl peptide receptor 2/lipoxin A_4_ receptor (FPR2/ALXR), via several signaling pathways, including the p38/mitogen-activated protein kinase (MAPK)-activated protein kinase (APK)/heat shock protein 27 (HSP27), c-Jun N-terminal kinase (JNK), and phosphatidylinositol 3-kinase (PI3K) pathways (Cooray et al., 2013), depending on the cell type. In monocytes and macrophages, LXA_4_ triggers the synthesis of IL-10 (Souza et al., 2007), a cytokine responsible for driving the resolution of inflammation, and enhances non-phlogistic phagocytosis of apoptotic cells (Maderna et al., 2010). Also, some effects of LXA_4_ are related to its ability to activate the cytosolic aryl hydrocarbon receptor (AhR), inducing the expression of suppressor of cytokine signaling (SOCS) (Machado et al., 2006).

The action of Resolvin E1 (RvE1) is intricate because it is an agonist of the seven-pass transmembrane GPCR ChemR23. Naturally, the activation of ChemR23 by low concentrations of chemerin, its natural agonist, favors chemoattraction of monocytes/macrophages and immature dendritic cells (DCs). Activation of ChemR23 increases intracellular calcium release and inhibits cAMP and MAPK extracellular signal-regulated protein kinases 1/2 (ERK1/2)-mediated signaling, by Gi/o protein (a G protein subtype) recruitment. Consequently, it leads to up-regulation of the PI3K/Akt signaling pathway and down-regulation of nuclear factor kappa-light-chain-enhancer of activated B cells (NF-κB) (Mariani and Roncucci, 2015). RvE1, via a different set of G proteins, stimulates Akt phosphorylation and ribosomal protein S6 kinase (Ohira et al., 2010). Also, RvE1 is a partial agonist of the LTB_4_ receptor 1 (BLT1) and competes with LTB_4_ for binding (Arita et al., 2007).

RvD1 can activate FRP2/ALXR and the G protein-coupled receptor 32 (GPR32) (Krishnamoorthy et al., 2012). The activation of the FPR2/ALX receptor by RvD1 suppresses cytosolic calcium and decreases activation of the calcium-sensitive kinase calcium-calmodulin-dependent protein kinase II (CaMKII). CaMKII inhibition suppresses activation of p38 and the MAP kinase-APK 2 (MAPK-APK2), which reduces Ser^271^ phosphorylation of 5-LO and shifts 5-LO from the nucleus to the cytoplasm (Fredman et al., 2014). Moreover, LXA_4_ and RvE1 counter-regulate the LTB_4_/LL-37 proinflammatory circuit, which is partially mediated by FPR2/ALX (Wan et al., 2011).

### Physiologic Actions of SPMs

LXA_4_ decreases polymorphonuclear leukocyte (PMN)-mediated tissue damage, angiogenesis, and PMN proliferation and adhesion, increasing non-phlogistic phagocytosis and IL-10 production (Chandrasekharan and Sharma-Walia, 2015). RvE1 increases PMN apoptosis, LXA_4_ production, and microbial killing, and decreases IL-12 production, PMN transendothelial migration, and PMN infiltration; also, it inhibits NF-κB reporter gene activation and reduces organ fibrosis (Arita et al., 2007; Campbell et al., 2007; El Kebir et al., 2012). RvD1 decreases reactive oxygen species (ROS) generation and the production of pro-inflammatory cytokines. RvD1 also reduces organ fibrosis (Qu et al., 2012; Miyahara et al., 2013). RvD2 increases microbial killing and clearance, as well as the production of nitric oxide (NO•) and PGI_2_ in endothelial cells (Spite et al., 2009; Chiang et al., 2012). PD1 decreases COX-2 expression, T-cell migration, and cytokine production, including that of TNF, IFN-γ, and microglial cell cytokines (Ariel et al., 2005; Qu et al., 2015). Also, PD1 has neuroprotective actions (Serhan et al., 2015). Maresins increase tissue regeneration, stimulating phagocytosis and the killing of oral pathogens by human leukocytes, and also promote the macrophage phenotype switch from M1 to M2 (Wang et al., 2015a). It is important to emphasize that both Rvs and LXs promote bacterial clearance; thus, they both constitute a pathway that impedes microorganism evasion of the immune response. Thus, the concert of the SPMs in the inflammatory broth give a change of helm heading toward a calmer environment, where leukocyte and macrophage hyperactivity decline, promoting a favorable environment for the repair.

### Role of SPMs in Chronic Infectious Diseases

Infections may progress toward chronicity due to dysregulation of the inflammatory process. Thus, extensive damage may occur involving irreversible structural alterations. Furthermore, it has been shown that in chronic inflammatory states such as Alzheimer’s disease (Wang et al., 2015b), periodontitis (Van Dyke, 2017), peripheral artery disease (Miyahara et al., 2013), or cystic fibrosis (Karp et al., 2004), the levels of pro-resolving factors such as LXA_4_, or ATLs are decreased. Therefore, it is suggested that a deficit of resolutory ability promotes chronic inflammatory states. Evidence of the participation of lipid pro-resolving mediators in infectious diseases is abundant. For example, in experimental models of lung infections (e.g., disseminated influenza A), there is low expression of LX (Cilloniz et al., 2010), and exogenous administration of PD1 improves survival (Morita et al., 2013). Moreover, when inflammation persisted in a respiratory syncytial virus lung infection, and exogenous administration of LXs or Rvs reverses the inflammation (Shirey et al., 2014). In a self-resolving murine model of *E. coli* pneumonia, exogenous AT-RvD1 administration enhanced the clearance of *E. coli* and *Pseudomonas aeruginosa in vivo*, and lung macrophage phagocytosis *ex vivo* (Abdulnour et al., 2016). These findings were similar to the results of a lung coinfection model with *Streptococcus pneumoniae* and influenza A virus (Wang et al., 2017), which showed that AT-RvD1 decreased the inflammatory drive by acting on the FPR2/ALX receptor and antagonizing the effect of serum amyloid A, which is an agonist of this receptor. Thus, there is a clear relationship between bacterial or viral infection and SPMs generation.

However, the LXs-bacterial interplay in a chronic infection may be more complicated. In a mouse model of *Mycobacterium tuberculosis* infection, 5-LO knockout mice were more likely to survive compared with wild-type mice, which had a more protracted disease evolution. Administration of LXA_4_ inhibited the development of a Th1 response, which is protective in the tuberculosis model (Bafica et al., 2005). Bacteria may also inhibit the resolution of infectious processes. A recent report demonstrated that chronic *P. aeruginosa* infections in cystic fibrosis patients promote the hydrolysis of an endogenous epoxide-containing eicosanoid 14,15-epoxyeicosatrienoic acid (14,15-EET) to its corresponding diol, thereby destroying the signal that triggers increased biosynthesis of 15-epi-LXA_4_ and preventing the activation of resolution pathways that could help to eradicate the infection (Flitter et al., 2017).

Specialized pro-resolving mediators may contribute to eradicate infections and terminate the inflammatory input, as it is suggested by murine sepsis models, where there was reported that LXA_4_ decreased plasma IL-6, chemokine (C–C motif) ligand 2 (CCL2), IL-10, and NF-κB activity in peritoneal macrophages, reduced neutrophil migration, and increased the clearance of bacteria by neutrophils without the production of excessive free radicals (Walker et al., 2011; Wu et al., 2015). Furthermore, LX may increase the production of antibacterial proteins. Bactericidal/permeability-increasing protein (BPI) is increased in an *in vitro* model of *Salmonella typhimurium* infection when LXs are administered (Canny et al., 2002). Thus, LX may have a role in the clearance of pathogenic microorganisms. Interestingly, LXA_4_ decreases the release of the exotoxin pyocyanin by *P. aeruginosa*, reducing its pathogenicity (Wu et al., 2016). Thus, LXA_4_ also affects infective agents. Furthermore, RvD1 and RvD5 were shown to decrease the dose of antibiotics needed to treat *E. coli* or *Staphylococcus aureus* infections (Chiang et al., 2012). Thus, RvD1 and RvD5 can help to clear bacteria and, most importantly, this research provides new insight into how to circumvent antibiotic resistance (Chiang et al., 2012).

In addition to its action in bacterial infections, LXA_4_ plays a role in acute and chronic parasitic infections. However, it is controversial because in murine models of cerebral malaria, LXA_4_ is associated with a lower parasite burden, less cerebral inflammation, and better survival (Shryock et al., 2013). However, in a murine model of *Toxoplasma gondii* infection, although LXA_4_ production is increased and there is less cerebral inflammation (due to decreased IL-12 levels), the immune response against the parasite is dampened (Aliberti et al., 2002). Probably, this despair results are due to the distinct causative organism or by the inflammatory context where LXA_4_ was produced.

## *Trypanosoma cruzi* Infections

*Trypanosoma cruzi* is a flagellate protozoan that causes Chagas disease (CD). The life cycle of the parasite includes survival inside muscle cells, including cardiac muscle and smooth muscle of the gastrointestinal tract (Rassi et al., 2012). The infection process involves an intense inflammatory response, which is coordinated by pro-inflammatory mediators such as PGs (Celentano et al., 1995; Cardoni and Antunez, 2004), leukotrienes, cytokines, and chemokines that increase the expression of endothelial cell adhesion molecules (ECAMs), allowing the migration of immune cells to the infection site (Golias et al., 2007). The increase in ECAMs induces vascular permeability and leukocyte recruitment (Gomes et al., 2014). These events are pivotal in the pathogenesis of chronic Chagas cardiomyopathy (CCC, the most lethal form of CD) because they facilitate leukocyte adhesion to cardiac endothelial cells and cause endothelial dysfunction. In turn, endothelial dysfunction is associated with focal ischemic events and microvascular abnormalities. Additionally, in CCC, the observed microvascular damage is worsened by platelet aggregation, which is activated by thromboxane A_2_ (TXA_2_), promoting a procoagulant environment that could exacerbate the focal ischemia (Cardoni and Antunez, 2004; Abdalla et al., 2008; Rossi et al., 2010; Prado et al., 2011). Thus, if left untreated, low-grade myocarditis initially ensues and the infection progresses from the acute to the chronic stage without necessarily involving clinical manifestations. As the infection and endothelial dysfunction persist, a myocardial remodeling process is launched, establishing CCC. Consequently, 30% of infected individuals develop cardiac complications, which can induce death by heart failure (Rassi et al., 2012; Ribeiro et al., 2012).

### Role of SPMs in Acute Chagas Disease

Among the acute inflammatory mediators, AA derivatives have been described as essential drivers of the acute infection process and the chronic cardiac damage induced by the parasite. Experimental *in vivo* models of infection with *T. cruzi* showed an increase in the expression of COX-2 (Molina-Berrios et al., 2013a) and PGs E receptor 2 (EP_2_) in cardiac tissue (Guerrero et al., 2015). These proteins are involved in the synthesis and activity of AA derivatives, such as PGE_2_, TXA_2_, PGF_2α_, 6-oxo-PGF_1α_, LTB_4_, and other eicosanoids that have been observed increased after *T. cruzi* infection. Consequently, cells derived from *T. cruzi*-infected mice shows elevated levels of PGE_2_ (Celentano et al., 1995; Borges et al., 1998; Freire-de-Lima et al., 2000; Guerrero et al., 2015). Also, PGE_2_, TXA_2_, PGI_2_, PGF_2α_, and LTB_4_ levels have been found increased in plasma of mice infected with *T. cruzi* (Cardoni and Antunez, 2004; Molina-Berrios et al., 2013a,b; Sharma et al., 2013). Also, it is well-documented that *T. cruzi* itself can synthesize AA derivatives like TXA_2_, PGE_2_, and PGF_2α_ by the action of the *T. cruzi* old yellow enzyme (TcOYE) (Kubata et al., 2002; Ashton et al., 2007). There is evidence that both, host- and parasite-derived autacoids contribute importantly to cardiac remodeling. In fact, COX-2, PLA2γ, or 5-LO deficient mice, infected with *T. cruzi*, exhibited improved survival rate and reduced cardiac tissue inflammation when compared with wild-type mice (Celentano et al., 1995; Borges et al., 1998; Cardoni and Antunez, 2004). Furthermore, there is evidence indicating that *T. cruzi* induces the formation of lipid bodies, specialized organelles where PGs synthesis occurs, being an important strategy for parasite growth and survival (Melo et al., 2003; D’Avila et al., 2011). The effect of the host or parasite-derived AA derivatives in the acute infection onset has been reviewed extensively elsewhere (Machado et al., 2011).

### 15-D-PGJ_2_ Is Pro-resolving in *T. cruzi* Infection

An aspect of growing interest is the implication of the PPAR-γ pathway in the modulation of inflammatory processes in chronic infections (Kim et al., 2015). In this regard, this pathway is related to the decrease in the transcription of genes controlled by NF-κB and the activator protein-1 (AP-1). It is well known that 15-D-PGJ_2_, which is a pro-resolving lipid derived from PGD_2_, acts as an agonist of PPAR-γ (Paumi et al., 2004). Therefore, it may have a role in pro-resolving processes in conjunction with other SPMs. Consequently, 15-D-PGJ_2_ has been tested as a modulator of acute and chronic heart inflammation in *T. cruzi* infection. 15-D-PGJ_2_ (1 mg/kg) decreases the inflammatory infiltrate and amastigote nest count and significantly increases the IL-10 levels (Rodrigues et al., 2010). Also, 15-D-PGJ_2_ attenuates acute liver damage induced by *T. cruzi* in mice. In this model, 15-D-PGJ_2_ was able to decrease fibrosis and liver damage without influencing the course of the infection itself (Penas et al., 2016). Moreover, a preliminary report from the same group suggested that treatment with 15-D-PGJ_2_ could regulate the number of intracellular amastigotes in the cardiac tissue via PPAR-γ-dependent and PPAR-γ-independent pathways (Penas et al., 2013).

### Acetylsalicylic Acid in Acute and Chronic Chagas Disease

Due to the role of AA derivatives in *T. cruzi* infection, inhibition of COX activity has been proposed as a strategy for controlling parasite-induced disease. Although several non-steroidal anti-inflammatory drugs (NSAIDs), such as indomethacin, meloxicam, and celecoxib, have been assayed in mouse models of *T. cruzi* infection (Freire-de-Lima et al., 2000; Michelin et al., 2005; Abdalla et al., 2008; Hideko Tatakihara et al., 2008), the most studied NSAID is ASA, also known as aspirin. Interestingly, the effect of ASA seems to be mouse-species dependent. When *T. cruzi*-resistant mice (C57BL/6 or CD-1) are treated with ASA, parasitemia and mortality increases (Michelin et al., 2005; Hideko Tatakihara et al., 2008; Mukherjee et al., 2011). On the other hand, Balb/c mice, which are sensitive to *T. cruzi* infection, become healthy when ASA is administrated (Freire-de-Lima et al., 2000; Paiva et al., 2007; Hideko Tatakihara et al., 2008). Importantly, C57BL/6 mice generate more NO• than Balb/c mice after *T. cruzi* infection, and ASA treatment induces NO• production in Balb/c mice infected with *T. cruzi* (Hideko Tatakihara et al., 2008). Furthermore, in murine macrophages infected with *T. cruzi*, the inhibition of NO• synthesis partially prevents the effect of ASA (Lopez-Munoz et al., 2010), suggesting an important role for this mediator in the ASA effect.

More importantly, the effect of ASA has an important relationship with its dose. Most studies investigating ASA have been performed using a fixed dose of 25–50 mg/kg. However, studies using higher doses (>75 mg/kg) have shown no effect, or they have shown that ASA aggravates the damage caused by intraperitoneal or oral *T. cruzi* infection (Molina-Berrios et al., 2013a; Cossentini et al., 2016). The fact that ASA has an antichagasic effect only at doses <50 mg/kg has been associated to the production of 15-epi-LXA_4_, an ASA-triggered LX found in patients treated with low doses of ASA (Chiang et al., 2004). In Balb/c *T. cruzi*-infected mice, 25 and 50 mg/kg of ASA induced a significant increase in 15-epi-LXA_4_ production without modification of LTB_4_ levels. At these doses, the mice had prolonged survival, decreased mortality, and less cardiac inflammatory infiltrate. Additionally, the administration of 25 mg/kg exogenous 15-epi-LXA_4_ significantly decreased the parasitemia peaks and cardiac parasite load, improved the survival of the infected mice, and partially reversed the detrimental effect of high-dose ASA (Molina-Berrios et al., 2013a). Low doses of ASA also improve the vascular reactivity of mice infected with *T. cruzi*. Molina-Berrios et al. (2013b) evaluated the effect of ASA at 2 and 40 mg/kg and found that both regimens decreased ECAM expression and the TXA_2_ level. Also, 2 mg/kg/day ASA reduced the inflammatory infiltrate in mice hearts and improved the cardiac histology at 90 days post infection. Furthermore, in *in vitro* cardiac cells infected with *T. cruzi*, low doses of aspirin increased IL-1β and NO• release, and decreased transforming growth factor (TGF)-β release, and these effects disappeared when ASA concentrations were increased (Malvezi et al., 2014b).

Consistently, ATLs not only participate in the resolution of the damage produced by *T. cruzi* but also in the clearance of the pathogen. In an *in vitro* model of *T. cruzi*-infected macrophages, the addition of 0.3–1.25 mM of ASA significantly decreased the internalization of *T. cruzi* without altering macrophage viability (Carvalho de Freitas et al., 2017). Also, it has been reported that 0.625–2.5 mM of ASA decreased the internalization of *T. cruzi* and increased the release of IL-15 and NO•. However, co-administration of celecoxib (a COX-2 selective inhibitor) reverted the ASA effect and restored the invasive capacity of trypomastigotes, suggesting that functional COX-2 is necessary for the ASA effect. Furthermore, Boc-2 (a specific antagonist of the FRP2/ALX receptor) prevented the ASA effect, suggesting that the inhibition of invasion depends on the synthesis of 15-epi-LXA_4_ (Malvezi et al., 2014a).

The effects of other SPMs triggered by ASA have been studied. Ogata et al. (2016) evaluated the effect of AT-RvD1 on peripheral mononuclear cells (PBMCs) from patients with Chagas heart disease at stage B1, that is, with few heart abnormalities. Stimulation of PBMCs with a *T. cruzi*-derived antigen increased the production of INF-γ, TNFα, IL-10, and IL-13, while the pre-treatment of PBMCs with AT-RvD1 (100 nM) significantly reduced the production of INF-γ, with no changes in TNFα, IL-10, and IL-13. As INF-γ polarizes the immune response to a type Th1 response, and a low level of IL-10 indicates loss of the Treg response, both phenomena could be associated with the development of heart damage in CD. Therefore, decreasing INF-γ using AT-RvD1 has a beneficial role in chagasic cardiomyopathy. Furthermore, AT-RvD1 was able to reduce the percentage of necrotic PBMCs and their proliferation after stimulation with a *T. cruzi* antigen (Ogata et al., 2016).

Recently, an elevated plasma level of RvD1 has been found in *T. cruzi*-infected CD-1 mice. Also, RvD1, RvD5, and RvE2 (but not LXs, maresins, or PDs) were found in the lysates of *T. cruzi* trypomastigotes. Interestingly, there were no Rvs found in the lysates of *T. cruzi* epimastigotes or other protozoan parasites such as *T. gondii*, suggesting that the trypomastigotes themselves synthesize these SPMs (Colas et al., 2018). However, there was no direct evidence for a metabolic switch involving TcOYE or a yet unknown enzymatic activity that could explain a *T. cruzi* origin of these SPMs. There is currently no evidence of a *T. cruzi* 5-LO enzyme or another enzyme for SPM synthesis.

### Statins and SPMs in Chronic Chagas Disease

15-epi-LXA_4_ can also be produced by statins. Birnbaum et al. (2006) showed that statins stimulate 15-epi-LXA_4_ release from myocardial tissue. This effect of statins over COX-2 could be mediated by the overexpression of inducible NO• synthase (iNOS), which in turns nitrosilates COX-2 at the Cys^526^ residue, giving COX-2 the ability to generate the 15R-HETE intermediate metabolite, which is cleaved by 5-LO to generate 15-epi-LXA_4_ (Kim et al., 2005). This 15-epi-LXA_4_ production also requires that 5-LO remains in the cytoplasm. Thus, 5-LO, phosphorylated by protein kinase A (PKA) is attached to the nuclear membrane, where it is committed to leukotriene synthesis (Ye et al., 2008).

In *T. cruzi*-infected human endothelial cells, simvastatin induces the synthesis of 15-epi-LXA_4_ and decreases the ECAM expression induced by the parasite. This effect was reversed by the addition of AA-861 (a 5-LO inhibitor) and replicated when using exogenous 15-epi-LXA_4_. Interestingly, 15-epi-LXA_4_ inhibited NF-κB pathway activation, decreasing the phosphorylation of the NF-kappa-B inhibitor (IκB) and the IκB kinase (IKK), and preventing NF-κB p65 nuclear translocation. Thus, the action of 15-epi-LXA_4_ in *T. cruzi*-infected endothelial cells involves the NF-κB signaling pathway (Campos-Estrada et al., 2015). In addition, in a murine model of CCC, 40 mg/kg/day simvastatin decreased endothelial activation, inflammatory infiltration, and fibrosis in heart tissue, an effect that persisted for a long time after treatment stopped. When simvastatin was administered to 5-LO-deficient mice, the anti-inflammatory effect was not observed unless exogenous 15-epi-LXA_4_ was also administered. Thus, there is an association between simvastatin administration, 15-epi-LXA_4_ production, and cardiac improvement. Furthermore, 15-epi-LXA_4_ was still detectable 30 days post administration, suggesting that 15-epi-LXA_4_ is stable in serum and is associated with the observed sustained effects (Gonzalez-Herrera et al., 2017).

**Table 1** summarizes the experimental evidence showing the effect of pro-resolving mediators in *T. cruzi* infections *in vivo*. The findings support the idea that ASA and simvastatin have a positive impact on the resolution pathways, producing novel pro-resolving lipids such as 15-epi-LXA_4_ or AT-RvD1, modulating the inflammatory response, and decreasing ECAMs, leukocyte recruitment, and inflammatory infiltration. Also, the SPMs increase the survival rate in animal models of CD.

**Table 1:** Experimental evidence on the role of pro-resolving mediators in Chagas disease.

Pro-resolving mediator	Experimental model	Beneficial role in Chagas disease	Reference
15-epi-LXA_4_ (ASA-triggered)	Chronic model of Chagas cardiomyopathy	↑ Survival ↓ Cardiac parasite load ↓ Number of amastigote nests ↓ Inflammatory infiltration	Molina-Berrios et al., 2013a
	Peritoneal macrophages infected with *T. cruzi*	↓ Internalization of *T. cruzi* into macrophages	Malvezi et al., 2014a
15-epi-LXA4 (simvastatin-triggered)	Endothelial cells infected with *T. cruzi*	↓ CAM expression ↓ Cellular recruitment Effect dependent on NF-κB pathway	Campos-Estrada et al., 2015
	Chronic model of Chagas cardiomyopathy	↓ CAM expression ↓ Inflammatory infiltration and fibrosis ↓ Cardiac parasite load Effect dependent on 5-LO	Gonzalez-Herrera et al., 2017
AT-Resolvin D1	PBMC from patients with Chagas disease	↓ INF-γ ↓ Necrotic cells ↓ Proliferation	Ogata et al., 2016
15-D-PGJ2	Acute model of mice infected with *T. cruzi*	↓ Number of amastigote nests ↓ Inflammatory infiltration ↑ IL-10 ↓TNF-α, IL-6, IL-1β, and NF-κB activation ↓ Liver fibrosis, CTGF, and TGF-β	Rodrigues et al., 2010; Penas et al., 2013, 2016

*CAM, cellular adhesion molecules; 5-LO, 5-lipoxygenase; INF-γ, interferon-γ; TNF-α, tumor necrosis factor-α; IL, interleukin; NF-κB, nuclear factor kappa-light-chain-enhancer of activated B cells; PBMC, peripheral blood mononuclear cell; CTGF, connective tissue growth factor.*

## *Leishmania* spp. INFECTIONS

*Leishmania* spp. infections have become a paradigm to explain how the balance between Th1 and Th2 immune responses can effectively fight an intracellular parasite. In these infections, the Th1 response (mediated by TNF-α, IL-2, IL-12, and IFN-γ) exerts a protective role, while the Th2 response (mediated by IL-4, IL-5, and IL-10) is known as a disease promoter (reviewed in Maspi et al., 2016). The clinical manifestations of leishmaniasis are divided into three forms: cutaneous, mucocutaneous, and visceral leishmaniasis (VL).

### Cutaneous Leishmaniasis

Cutaneous leishmaniasis (CL) is the most common form of leishmaniasis around the world. CL is characterized by self-limiting skin lesions located in body areas where sandflies usually bite, such as the face, neck, and limbs. The disease progression varies between different world regions. The old-world CL (caused by *Leishmania major* or *Leishmania tropica*) progresses over weeks to months to form a dry ulcer, but healing occurs over several months or years, leaving a scar or depigmentation of the skin. On the other hand, the new-world CL (caused by *Leishmania mexicana, Leishmania amazonensis*, or several parasites from the *Vianna* subspecies, such as *L. [V] braziliensis*) produces a wet ulcer that is associated with lymphadenitis or lymphadenopathy and may involve mucosal manifestations. New-world leishmaniasis is also called tegumentary leishmaniasis (TL), which can be localized or disseminated (de Vries et al., 2015).

**FIGURE 2:**
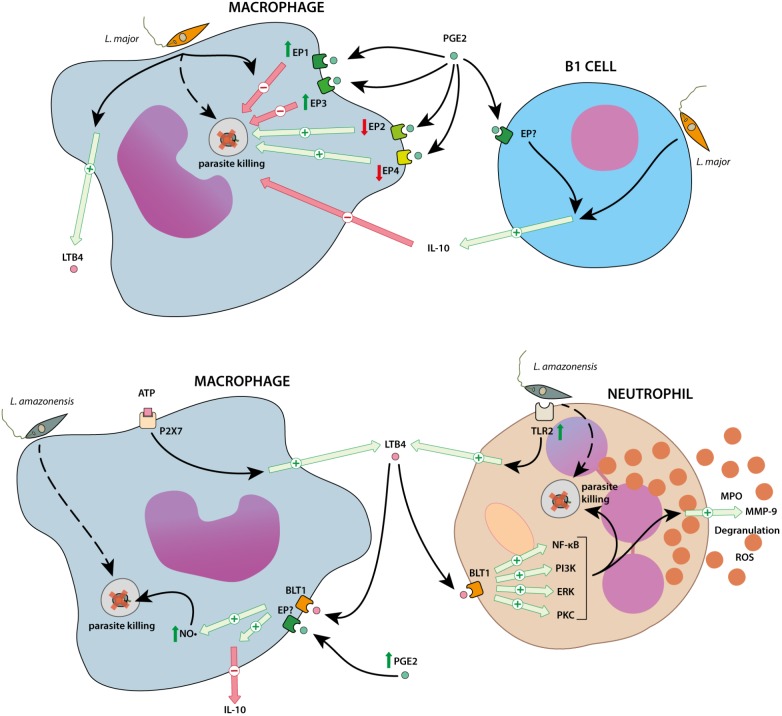
Overview of the regulation of inflammatory arachidonic acid (AA) derivatives after *Leishmania major* or *Leishmania amazonensis* infections. Cutaneous leishmaniasis causative parasites (*L. major* and *L. amazonensis*) induce the production of AA derivatives with opposite roles, depending on the *Leishmania* species. In *L. major* infection models (upper panel) the parasite induces the production of PGE_2_ and LTB_4_. Also, the expression of the EP_1_ and EP_3_ receptor is increased. The activation of these receptors by PGE_2_ leads to parasite survival. PGE_2_ also increases the release of IL-10 by the B1 cells, reducing the phagocytic activity of macrophages. On the other hand, *L. amazonensis* (lower panel) phagocytosis and killing by macrophages is increased by LTB_4_ and PGE_2_, and this effect is mediated by the NO• increase. Also, *L. amazonensis* increase the degranulation of neutrophils, increasing the anti parasitic activity of the immune system.

In patients with TL, there is a different pattern of expression of the genes involved in the AA cascade between patients with localized CL (LCL) and mucocutaneous leishmaniasis (MCL). Patients with MCL (the most inflammatory form, with a lower parasite burden per lesion) have decreased expression of the genes for PGE_2_ synthesis enzymes (*PTGS1* and *PGES*), whereas the expression of the gene for 5-LO (*ALOX5*) is increased. This divergent expression pattern correlates with the decreased levels of PGE_2_ and increased levels of LTB_4_ found in patients with MCL. Also, in this study, patients with MCL have increased expression of *PTGER3*, the gene coding for the PG receptor EP_3_ (Franca-Costa et al., 2016). This finding suggests a role for LTB_4_ in MCL-induced inflammation. However, studies on the role of the inflammatory AA derivatives (such as PGE_2_ or LTB_4_) at the molecular level show that this effect is highly *Leishmania* species-dependent (**Figure 2**).

In the 1980s, it was reported that higher levels of PGE_2_ relate to reduced lymphocyte proliferation in the spleens of mice infected with *L. major* (Farrell and Kirkpatrick, 1987). Moreover, the increase in PGE_2_ and LTB_4_ levels induced a reduction in the Th1 cytokines TNF-α and IFN-γ, and an increase in the Th2 cytokine IL-4. This effect has been linked to the overexpression of the PG receptors EP_1_ and EP_3_ (Milano et al., 1996). Indeed, in macrophages from Balb/c mice infected with *L. major*, there is an increase in these receptors and downregulation of EP_2_ and EP_4_ receptors. Furthermore, agonists of EP_1_ and EP_3_ favor infection, whereas selective agonists of EP_2_ and EP_4_ decrease infection (Penke et al., 2013). PGE_2_ also affects the role of B-1 cells. B-1 cells modulate the phagocytic activity of macrophages; this action is dependent on IL-10 release, which in turn modulates the PGE_2_ levels in the media (Arcanjo et al., 2017). Also, PGE_2_ (via IL-10 production) increases the phagocytic activity of Balb/c mice-derived B-1CDP cells (a particular B-1 cell type with phagocytic ability; Borrello and Phipps, 1999), but it impairs the ability of the mice to resist the infection (Arcanjo et al., 2015). Finally, in a model involving human PMNs co-stimulated with ionomycin or LPS/N-formyl-methionyl-leucyl-phenylalanine (LPS/fMLP), *L. major* was also able to increase LTB_4_ release, which in turn induced a rapid and sustained decrease in LXA_4_ production (Plagge and Laskay, 2017).

In contrast, in mouse models of *L. amazonensis* infection, higher levels of LTB_4_ and PGE_2_ enhance the parasite-killing ability of murine macrophages, an effect that is dependent of NO• production and IL-10 reduction (Guimaraes et al., 2006; Serezani et al., 2006). The high levels of LTB_4_ from macrophages can also be induced by ATP, via P2X7 receptor activation (Chaves et al., 2014). LTB_4_ also has a role in neutrophil-dependent *L. amazonensis* killing. LTB_4_ activates its BLT_1_ receptor, inducing neutrophil degranulation, the release of metalloproteinase 9 (MMP-9) and myeloperoxidase (MPO), ROS production, and overexpression of TLR2. All these phenomena may be associated with the activation of NF-κB, PI3K, ERK, and protein kinase C (PKC) signaling (Tavares et al., 2014).

**Table 2** resumes all current available evidence regarding the role of pro-resolving lipids in *Leishmania* spp. infections. Of note, most of the researches have been focused in CL. Regarding the role of these pro-resolving lipids in CL, RvD1 increases in patients with diffuse CL (DCL), in comparison with those with LCL, who had lower levels of RvD1 and higher levels of RvD2. This RvD1 pattern in DCL patients correlates with higher levels of Arginase-I and TGF-β and lower levels of TNF-α (Malta-Santos et al., 2017). In an *in vitro* model of *Leishmania* infection based on human cells, RvD1 increased phagocytosis of the *Leishmania* parasites by human macrophages. The increase of RvD1 in *L. amazonensis*-infected macrophages is reversed by baicalein, an inhibitor of 15-LO (Malta-Santos et al., 2017).

**Table 2:** Experimental evidence on the role of pro-resolving mediators in *Leishmania* spp infections.

Leishmania-induced disease	Host/infection model	Pro-resolving lipid/receptor	Associated inflammatory markers/outcomes	Reference
Cutaneous/mucocutaneous leishmaniasis	DCL patients serum	↑ RvD1 and ↓ RvD2	↑ Arginase-I and TGF-β ↓ TNF-α ↑ Number of lesions	Malta-Santos et al., 2017
	LCL patients serum	↓ RvD1 and ↑ RvD2	↓ Arginase-I ↑ TNF-α Low number of lesions	Malta-Santos et al., 2017
	*Ex vivo* model of infection with *L. amazonensis*	RvD1 treatment	↑ Leishmania phagocytosis by human macrophages.	Malta-Santos et al., 2017
	C57BL/6 mice (KO for AhR receptor) infected with *L. major*	↓ LXA4	↑ TNF-α ↓ IL-12	Elizondo et al., 2011
	Human PMNs infected with *L. major*	↓ LXA4	↑ LTB4	Plagge and Laskay, 2017
	Balb/c mice infected with *L. braziliensis* and serum of ML patients	↑ Annexin A1	↑ NF-κB phosphorylation	Oliveira et al., 2017
Visceral leishmaniasis	Balb/c mice and golden hamster infected with *L. donovani*	15-D-PGJ2 treatment	↓ Parasite load ↓ IL-10 and TGF-β ↑ TNF-α and IL-12	Vishwakarma et al., 2016

*DCL, diffuse cutaneous leishmaniasis; LCL, localized cutaneous leishmaniasis; ML, mucocutaneous leishmaniasis; RvD1, Resolvin D1; RvD2, Resolvin D2; LXA4, Lipoxin A4; AhR, aryl hydrocarbon receptor; TNF-α, tumor necrosis factor-α; IL, interleukin; NF-κB, nuclear factor κ-light-chain-enhancer of activated B cells; PMN, polymorphonuclear leukocyte; 15-D-PGJ_2_, 15-deoxy-Δ(12,14)-prostaglandin J_2_.*

RvD1 and LXA_4_ also target AhR, a transcription factor activated by several tryptophan metabolites and some AA derivatives. AhR has been identified in Treg cells, Th17 cells, and DCs, and its activation induces the expression of IL-10 and TGF-β (Gutierrez-Vazquez and Quintana, 2018). In *L. major* infections, the deletion of this receptor accelerates the disease progression, increasing TNF-α production, and decreasing IL-12 and LXA_4_ synthesis (Elizondo et al., 2011).

Annexin A1 is a 37-kDa protein that is expressed under the control of glucocorticoids and activates FPR2/ALXR, increasing IL-10 and IL-6 in experimental models of inflammation (reviewed by Perretti and D’Acquisto, 2009). In mice infected with *Leishmania [V] braziliensis*, Annexin A1 expression is correlated with the lesion size, being higher 2 weeks after infection. Annexin A1 is important to control the inflammatory response while not impairing the immune system’s parasite-killing ability. Indeed, Annexin A1-KO mice display more phosphorylation of NF-κB after *L. braziliesis* infection. Also, Annexin A1 is increased in patients with the mucosal form of the disease, but not in those with the localized cutaneous form (Oliveira et al., 2017). In addition, the synthesis of LXA_4_, the primary agonist of FPR2/ALXR, is decreased in PMNs infected with *L. major* (Plagge and Laskay, 2017). LXA_4_, via FPR2/ALXR activation, enhances the phagocytic activity of PMNs in a dose- and time-dependent manner (Wenzel and Van Zandbergen, 2009).

### Visceral Leishmaniasis

**FIGURE 3:**
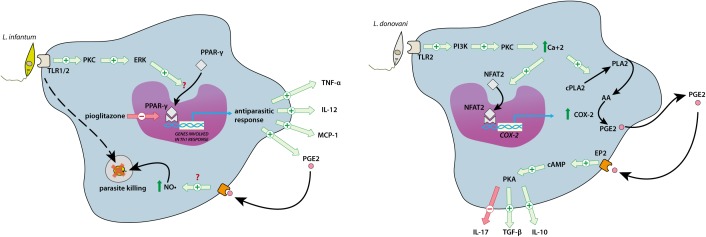
Overview of the regulation of inflammatory arachidonic acid (AA) derivatives after *Leishmania infantum* or *Leishmania donovani* infections. Visceral leishmaniasis causative parasites (*L. infantum* and *L. donovani*) induce the production of AA derivatives with opposite roles, depending of the *Leishmania* species. *L. infantum* infection (right panel) induce the release of TNF-α, IL-12, MCP-1 and PGE_2_ from macrophages. PGE_2_, in turn, induce the production of NO•, enhancing parasite killing. In contrast, *L. donovani* (left panel) increase the production of PGE_2_ by magrophages, through the activation of the PI_3_K pathway and the NFAT2 transcription factor. This augmented PGE_2_ activates the EP_2_ receptor in macrophages, inducing the release of regulatory cytokines such as TGF-β and IL-10, thus impairing the immune response against the parasite.

More than 30 years ago, it was reported that *Leishmania donovani* induces the synthesis of PGE_2_ and LTB_4_ in infected macrophages (Reiner and Malemud, 1984, 1985). The interaction of *L. donovani* with the TLR2 receptor in macrophages induces activity in the PI3K/Ca^+2^ axis, activating the cytosolic phospholipase A2 enzyme (cPLA2) enzyme, which release AA from membranes. Also, the Ca^+2^ increase activates the nuclear factor of activated T-cells (NFAT2), a transcription factor which translocates into the nucleus and induces the expression of COX-2. Both AA release and COX-2 overexpression are responsible for the resultant increased PGE_2_ release (Bhattacharjee et al., 2016). PGE_2_ is anti-inflammatory, leading to parasite survival via EP_2_ receptor activation. This EP_2_ activation triggers cAMP synthesis, with subsequent PKA activation. This second messenger cascade allows the release of IL-10 and TGF-β and reduces the levels of the inflammatory cytokines TNF-α, IL-12, and IL-17 (**Figure 3**). Consequently, the inhibition of COX-2 or EP_2_-mediated activation of PKA enhances the antiparasitic ability of immune cells *in vitro* and *in vivo* (Saha et al., 2014).

15-D-PGJ_2_, a pro-resolving lipid that acts as an activator of PPAR-γ receptors (Croasdell et al., 2015), decreases the parasite load *in vitro* and *in vivo* (**Table 2**). This antiparasitic activity correlates with a significant decrease in IL-10 and TGF-β, and a slight increase in the proinflammatory cytokines TNF-α and IL-12, suggesting that 15-D-PGJ_2_ favors the Th1 response against *L. donovani* (Vishwakarma et al., 2016).

Nevertheless, as in CL, the effect of AA derivatives is also *Leishmania* strain-dependent (**Figure 3**). *Leishmania infantum* is a parasite that infects a broad variety of dog populations, inducing canine VL (CVL). This parasite can also infect humans, thus dogs are a reservoir of the disease in large endemic areas of South America (Romero and Boelaert, 2010). Dogs infected with *L. infantum* show lower levels of PGE_2_ and LTB_4_ compared with uninfected animals. Moreover, lower levels of PGE_2_ and LTB_4_ correlate with an increase in the severity of CVL presentation (Solca et al., 2016).

At the cellular and molecular level, PGE_2_ increases parasite killing in macrophages infected with *L. infantum*; this effect is dependent on NO• release (Brandonisio et al., 2001; Panaro et al., 2001). Lymph node-derived leukocytes from dogs infected with *L. infantum* produce higher levels of NO•, TNF-α, and PGE_2_. Consequently, the pharmacological inhibition of PGE_2_ synthesis using indomethacin reduces TNF-α release from these cells (Venturin et al., 2016). This PGE_2_ release is stimulated by the activation of TLR1/2 receptors by the lipophosphoglycans of *L. infantum*, which induces the PKC-ERK1/2 pathway, causing the release of the Th1 cytokines TNF-α, IL-12, and monocyte chemoattractant protein-1 (MCP-1). In a dog model, PPAR-γ agonists, such as rosiglitazone, inhibit the cytokine storm induction. The fact that Th1 cytokine overexpression is associated with the inhibition of PPAR-γ (Lima et al., 2017) concurs with the fact that NSAIDs not only inhibit COX activity but can also activate this transcription factor (Lehmann et al., 1997), adding another possible mechanism of action for the antiparasitic activity of NSAIDs in *in vivo L. infantum* infections (**Figure 3**) (Panaro et al., 2001; Venturin et al., 2016). Conversely, in an *L. infantum*-resistant mouse model, the animals became sensitive when the activity of 5-LO was prevented. Spleen cells obtained from these mice showed that the IL-17-producing CD4+ T cells were significantly impaired, with a consequent reduction of cytokine release related to the Th17 axis (Sacramento et al., 2014).

In humans presenting with VL, the pattern of cytokines and lipids that drives the inflammatory response is diverse. In a cohort of patients from an endemic area in Brazil, patients with VL had elevated levels of IL-10, IL-6, IL-8, IL-12, RvD1, LTB_4_, and PGF_2α_. Also, these patients had lower levels of TGF-β1 and TNF-α. Moreover, this pattern of immune modulators reverted after treatment with antimonial compounds, increasing the levels of TGF-β1 and decreasing the levels of LTB_4_, RvD1, IL-6, IL-8, and IL-10. This study also showed that TNF-α levels were not modified by chemotherapy treatment, indicating that the inflammatory response to kill the parasite remained unaltered (Araujo-Santos et al., 2017).

## *Trypanosoma brucei* INFECTIONS

*Trypanosoma brucei* subsp. are responsible for human African trypanosomiasis (HAT), also known as sleeping sickness. Currently, HAT is the most neglected disease among the so-called Tri-Tryp (*T. brucei, T. cruzi*, and *Leishmania* spp.) and the volume of research on the inflammatory aspects of the disease are below those for other trypanosomatids. HAT progresses from a hemolymphatic early stage, which is characterized by the presence of parasites in the bloodstream, to a meningoencephalitic or late stage, where the parasite crosses the blood–brain barrier and causes an inflammatory encephalitic reaction that ultimately causes the death of the human host. The early stage (1–3 weeks) begins when the tsetse fly bites its host, depositing parasites held within its saliva on the human skin. Later, the parasites spread to various peripheral organs and tissues via the lymph and blood, inducing symptoms that include general malaise, anemia, weakness, and weight loss. The late stage coincides with the parasite invasion of the central nervous system (CNS), and it is associated with neurological alterations such as sleep disorders, confusion, and mental discoordination. Neuropsychiatric symptoms increase in frequency and severity with disease progression and untreated patients progress to a final stage involving seizures, drowsiness, coma, and death (Sternberg and MacLean, 2010; Büscher et al., 2017).

Although symptoms are common for both *T. brucei rhodesiense-* and *T. brucei gambiense*-associated HAT, the clinical presentation depends on which of the two subtypes of *T. brucei* is involved in the infection. *T. brucei gambiense* is associated with a slow-progressing form of HAT, whereas *T. brucei rhodesiense* is related to a faster-progressing form that can cause CNS damage within a few weeks of infection (Büscher et al., 2017). The interplay between the host immune response and parasite subspecies virulence patterns determines the progression and severity of the disease for each particular patient (Ponte-Sucre, 2016).

The initial response against the parasite mainly involves a Th1 pro-inflammatory cytokine profile including TNF-α, IL-6, NO•, IL-1, and IL-12 (Schleifer and Mansfield, 1993). At the same time, *T. brucei gambiense* activates a Th-dependent B-cell response against the main antigenic molecule of *T. brucei*, variant surface glycoprotein (VSG), allowing clearance of the organisms from the blood (Mansfield and Paulnock, 2005). As reviewed by Ponte-Sucre (2016), the presence of VSG allows the immune system to exert a lytic antibody response against *T. brucei*, but the parasite’s ability to switch to new VSG coats generates a parasite population that are not recognized by the previously generated antibodies. Although VSG is considered the primary antigenic molecule of *T. brucei*, parasite DNA is released into the plasma of infected mice, acting as a pathogen-associated molecular pattern (PAMP). This DNA activates macrophages in the first days post infection, increasing IL-12 levels, probably to induce a response to control the parasite levels by enhancing Th1 cell polarization (Sternberg et al., 2005). On the other hand, *T. brucei rhodesiense* DNA also increases IL-10 levels, which could play a role in controlling the immune response, as this cytokine has been shown to limit immunopathology (Sternberg et al., 2005; Harris et al., 2006).

Common to other trypanosome species, the success of *T. brucei* infection relies on its ability to overcome the initial immune system response but to an extent that is compatible with the life of the host, avoiding a devastating “hyper-infection” (Ponte-Sucre, 2016). In this respect, resolution of the inflammatory response is one of the main events that *T. brucei* modulates to escape the initial immune system attack. An early study showed that suppressor macrophages obtained from mice infected with *T. brucei* were able to inhibit production of IL-2 and the expression of the IL-2 receptor, decreasing T-cell activation but not pro-inflammatory secretion of IL-1, which could be produced by an increase in PGs (Sileghem et al., 1989). These effects are concordant with following studies reporting that suppressor macrophages control T-cell activation in *T. brucei* infection through NO• synthase (NOS) up-regulation and that elevated NO• produced by macrophages derived from infected mice is also dependent on PG synthesis (Schleifer and Mansfield, 1993). At that time, it was clear that PGs had a role in mediating the initial avoidance of the immune system as, in several cell types, *T. brucei* elicited an increase in these eicosanoids; the discovery of a parasite PGF_2α_ synthase further reinforced this idea (Alafiatayo et al., 1994; Kubata et al., 2000). In fact, administration of the classic COX inhibitor sodium salicylate (an ASA metabolite) to chronically *T. brucei*-infected Sprague-Dawley rats induced a marked increase in neurotoxicity, with an increase in mRNA levels of pro-inflammatory cytokines such as IL-1β, IL-6, and IFN-γ, and an increase in COX-2 and iNOS enzymes (Quan et al., 2000). This evidence suggests that, to some extent, PGs play a role not only in the acute phase of the infection but also in the late stage, probably controlling the parasite burden. In bloodstream forms of *T. brucei rhodesiense*, PGD_2_ and its metabolites can inhibit parasite growth and induce apoptotic-like programmed cell death through ROS generation (Figarella et al., 2005, 2006). Salmon et al. (2012) reported that *T. brucei* adenylate cyclases (ACs) play a role in reducing the early innate defense against live parasites by inhibiting TNF-α synthesis in infected mice. The authors demonstrated that cAMP-mediated activation of PKA affected trypanosome infection, an effect mediated by PKA signaling activation (Salmon et al., 2012). It is well known that PGs can activate cAMP-mediated responses in different cell types; hence, it is possible that these inflammatory mediators could act through these signaling pathways to control parasite survival, although there is no direct evidence for this postulation, at least regarding *T. brucei*.

Another relevant aspect is the control of inflammation and damage to different organs elicited by *T. brucei* in the late stage of the disease. In this respect, the regulatory cytokine IL-10 is induced by the parasite, decreasing the levels of NO• and TNF-α in infected mice, reducing organ damage, and favoring host survival (Guilliams et al., 2009). Also, results from several animal studies indicate that *T. brucei*-induced IL-10 production counters anemia; thus, this cytokine may play a crucial role in parasite and host survival (Musaya et al., 2015), which concurs with findings from research on human subjects. In the late stage, the levels of IL-10 were found to be elevated irrespective of the geographical location of the patients and the particular genotype of the strain involved in the infection (Maclean et al., 2004). In a more recent study, Kato et al. (2015) evaluated plasma and cerebrospinal fluid (CSF) cytokine levels in patients with early- or late-stage HAT. Although the authors did not find a difference in the levels of IFN-γ, TGF-β, IL-6, and IL-10 in the plasma, the CSF samples showed an up-regulation of IL-6 and IL-10 in the late-stage patients, which was associated with a reduction in severity of neurological impairment (Kato et al., 2015). In contrast, “trypanotolerant” individuals with elevated IL-10 levels and low levels of TNF-α are associated with a higher risk of developing HAT (Ilboudo et al., 2014). This evidence indicates that *T. brucei* can regulate both the inflammatory response in both the early and late stage of the infection, to ensure host survival. Although there is increasing evidence of inflammatory mediators such as cytokines and PGs, the “fine-tuning” of inflammation and parasite survival comprises a series of molecules and processes rather than a single mechanism.

## Concluding Remarks

Trypanosomatids in general and *T. cruzi* and *Leishmania* spp. in particular are responsible for chronic disabling and potentially fatal diseases. The understanding of the pathophysiological processes in which the parasite develops is fundamental for the design of much more effective therapies. In this regard, it is very promising to contemplate modifying aspects of the inflammation induced by the parasites in order to resolve or diminish the inflammation, promoting a more efficient clearance of the microorganism by the host’s immune system. There is growing evidence addressing this point. Therefore, the use of pro-resolving lipids, mainly ASA-triggered LXs and Rvs, together with antiparasitic therapy itself, could help to prevent the damage induced by the chronic inflammation generated by this group of parasites.

In this context, SPMs such as LXA_4_, RvD1, and drug-induced pro-resolving lipids (such as 13-epi-LXA_4_ and AT-RvD1) have been proven to be effective in the control of the inflammatory response against parasite infection in most animal models. However, the fact that the parasite itself could induce the release of these agents (or eventually synthesize them) indicates that the control of acute inflammation could also be beneficial for the parasite. Thus, the use of SPMs as a strategy against trypanosomatid infections should not be a universal consideration, as the effects of SMPs are highly variable between different parasite species/strains and even different mammals. Overall, there is a need for more research to elucidate which parasites, host conditions, or even infection stages are associated with safe and effective use (or synthesis stimulation) of these SPMs.

## Author Contributions

RL-M, AM-B, CC-E, PA-S, LU-L, MP-E, and JM performed the literature search, analyzed the articles, and wrote the manuscript. RL-M and JM made the figures. All authors approved the manuscript in its final version.

## Conflict of Interest Statement

The authors declare that the research was conducted in the absence of any commercial or financial relationships that could be construed as a potential conflict of interest.
